# The Role of Scent Marking in Mate Selection by Female Pumas (*Puma concolor*)

**DOI:** 10.1371/journal.pone.0139087

**Published:** 2015-10-21

**Authors:** Maximilian L. Allen, Heiko U. Wittmer, Paul Houghtaling, Justine Smith, L. Mark Elbroch, Christopher C. Wilmers

**Affiliations:** 1 Center for Integrated Spatial Research, Environmental Studies Department, University of California Santa Cruz, Santa Cruz, United States of America; 2 School of Biological Sciences, Victoria University of Wellington, Wellington, New Zealand; 3 Panthera, New York, NY, United States of America; University of Missouri, UNITED STATES

## Abstract

Mate selection influences individual fitness, is often based on complex cues and behaviours, and can be difficult to study in solitary species including carnivores. We used motion-triggered cameras at 29 community scrapes (i.e. scent marking locations used by multiple individuals) and home range data from 39 GPS-collared pumas (*Puma concolor*) to assess the relevance of communication behaviours for mate selection by female pumas in California. Female pumas visited community scrapes irregularly and visitation bouts appeared to be correlated with oestrus. Female pumas on average selected from 1.7 collared males, and selection was based on multiple cues that varied among the different time periods measured (i.e. the female’s visitation bout and in 90 days previous to the consorting event). Female mate selection over the course of a visitation bout was based on frequency of the male visitation, mass, and age. In the 90 days previous to consorting, the number of scrapes a male created was the most important contributor to selection, which was likely related to his residency status. We also found that at least 14% of females mated with multiple males, thus possibly confusing paternity. Our findings provide a mechanistic understanding of how female pumas use scent and auditory communication at community scrapes to select dominant resident males to mate with.

## Introduction

Reproductive success is essential for individual traits to transfer to, and hence modify, future generations [1], and therefore behaviours associated with reproduction are an important source of adaptation and evolution. How individuals select mates is complex and often involves multiple cues that vary among species [2,3], necessitating the development of species-specific behaviours and signals to advertise for and communicate with potential mates [4,5,6,7]. In order to understand the adaptive significance of behaviours used for mate selection, it is necessary to understand each cue and its associated function. However, the challenges associated with observing cues involved in mate selection of cryptic mammals such as solitary carnivores in natural environments have historically prevented detailed study of these behaviours [8,9].

Reproductive strategies in solitary carnivores are expected to vary between males and females. In these species, males are commonly polygynous and often significantly larger than females. Males are thought to compete with other males for territories that encompass the home ranges of multiple females and are believed to attempt to mate with every reproductive female residing within their territory [4,7,8,10,11]. Conversely, female solitary carnivores tend to overlap with few potential mates [8,12,13], and may primarily rely on carefully assessing cues to select a mate that best enhances survival of their offspring [2]. A key aspect of female mate selection is thought to be the body mass of potential mates. Larger mass in male carnivores appears to be selected for across generations as an indicator of greater fitness and physical dominance, both of which can be expressed via larger territories [8,9]. However, recent advances in our understanding of mate selection suggest that female selection is more complex than simplistic assessments of male ornamentation or mass [9].

The spatiotemporal dispersed structure of populations of solitary carnivores requires indirect methods to advertise for potential mates and communicate with competitors. Many solitary carnivores are thought to therefore communicate with competitors through indirect cues (i.e. scent marking and vocalizations) rather than through direct contact [8,13,14]. Felids are generally considered to have a poor sense of smell when compared to canids and ursids [8], but nevertheless, the most commonly observed form of indirect communication in solitary felids is scent marking [4,8,14]. Scent marking (e.g. via sprayed urine) potentially allows male felids to advertise their presence and residency status to other males and females whose home ranges they overlap [4,8,14,15]. Female solitary carnivores can then potentially visit scent marking areas used by multiple males and simultaneously assess potential mates.

Pumas (*Puma concolor*) are solitary, territorial felids, and primarily communicate through scent marking [8,16]. Pumas breed throughout the year [8,16], however, research suggests that parturitions in some areas peak in summer and early autumn [8,17,18]. For populations that experience peaks in parturitions, mating behaviour is also expected to peak (i.e. an additional 3 months earlier). Nevertheless, the potential for mating to occur throughout the year suggests that scent marking and other communication behaviours associated with mate selection, too, must occur throughout the year. Mate selection in pumas might be further complicated due to aggressive behaviours, including those causing injuries or death, of males towards females not in oestrus [8].

Scraping, where a puma uses its hind feet to create a mound of substrate material and then sometimes urinates or defecates on the mound (S1 Video), is the most frequently observed communication behaviour in pumas [8,19,20]. Following Allen et al. [20], we define scent marking areas where scrapes are concentrated or clumped (≥ 3 in 9 m^2^) as ‘community scrapes.’ Community scrapes are often used by multiple individual pumas, most frequently by resident males [20]. Pumas have been observed to display other behaviours at these scrape areas, including olfactory investigation, the flehmen response, and caterwauling (S2 Video) (Table 1) [20]. Male communication at community scrapes may be primarily to advertise for mates, but is also likely to deter competitors [20]. In contrast, the most frequent communication behaviours displayed by female pumas at community scrapes are investigative and may be used for the assessment of potential mates [20]. Although recent research has begun to explain how scraping and associated behaviours are used by pumas for intraspecific communication e.g., [8,19,20], we do not currently understand their functions in female mate selection.

**Table 1:** The definitions of behaviours exhibited by pumas at community scrapes, based on Allen et al. [20].

Behaviour	Definition
Caterwauling	A loud, reverberant call. Most frequently given by females.
Flehmen Response	Where the puma picked up its head and curled back its upper lip, sometimes arching its neck backwards, in order to expose its vomeronasal organ.
Olfactory Investigation	Where the puma is using its olfactory sense to investigate cues and signals, noted by the pumas nose within one head length of a scrape or other cue.
Scraping	Where the puma clawed in substrate with their hind feet and then sometimes urinated and/or defecated on the scraped mound of material.

We monitored a puma population that included 39 marked individuals (20 females and 19 males) in California from 2011–2013 to better understand female mate selection strategies. Pumas are difficult to observe due to their cryptic nature, so we deployed motion-triggered video cameras at known community scrapes to record communication behaviours, visitation rates, and to assess the relevance of communication behaviours for mate selection. We formulated the following three hypotheses: 1) Due to differences in mating strategies, patterns in visitation would vary between male and female pumas. We expected male pumas to visit regularly to advertise for females and mark for territorial ownership. We expected females to be irregular in visitation, avoiding community scrapes when raising kittens, and visiting when in estrus; 2) Because reproductive females generally mate once every two years, we expected that female pumas will make fewer forays to community scrapes outside of their core home ranges than males. Successful females generally mate once every two years, so less of their time is spent seeking out mates, and may be more likely to choose the resident male to improve safety of their dependent kittens from infanticide. In contrast, male pumas are expected to seek opportunities to mate with every available female, including making forays out of their home range to search for females, and being drawn outside of their home range by the caterwauling of females; 3) Females will select male pumas based on perceived residency (in order to avoid infanticide later), which males advertise by the frequency of their visitation to community scrapes. Because mass and age are related to residency in male pumas (Logan and Sweanor 2001), females may also indirectly select for those characteristics.

## Materials and Methods

### Study area

**Fig 1:**
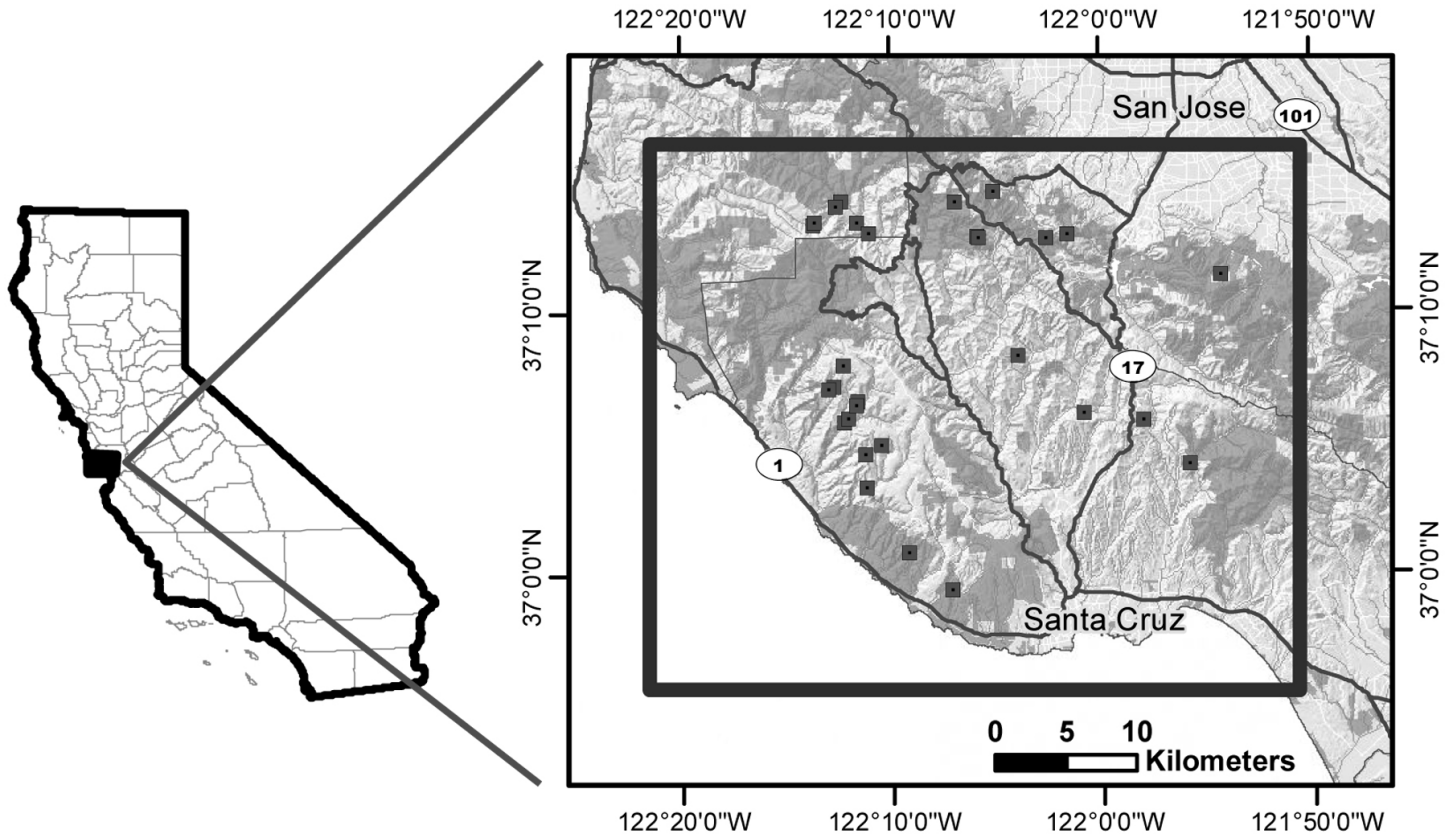
A map of the study area, showing the location of monitored community scrapes. The study area included areas in Santa Cruz, San Mateo, and Santa Clara Counties and the cities of Santa Cruz and San Jose in California.

Our study area is 1,700 km^2^ in the Santa Cruz Mountains of California, including parts of Santa Cruz, San Mateo, and Santa Clara counties (Fig 1). The study area is bounded by the Pacific Ocean to the west, the city of San Jose to the north, the city of Santa Cruz to the south and Highway 101 to the east. An arterial highway (California Highway 17) bisects the study area. Vegetation characteristics and climatic conditions in the study area have been described in detail elsewhere [20,21].

### Ethics statement

Our protocols for the capture of pumas adhered to the guidelines outlined by the American Society of Mammalogists [22], and were approved by the Institutional Animal Care and Use Committee of the University of California, Santa Cruz (Protocols Wilmc0709 and Wilmc1101), and the Wildlife Investigations Lab of the California Department of Fish and Wildlife. Puma capture and handling protocols have been described in Wilmers et al. [21], and no pumas were ever killed/sacrificed as part of research methods. Pumas were not endangered, and permission to handle pumas was granted through a Memorandum of Understanding and Scientific Collecting Permit #11968 with the California Department of Fish and Wildlife.

Our research was carried out on a combination of public and private land. In each case, we approached the property owner for permission to access their property before entering. Public properties that required permits were California State Parks, the city of Santa Cruz, Midpeninsula Regional Open Space District, Penninsula Open Space Trust, and Santa Clara County Parks.

### Field methods

We monitored behaviour of pumas at community scrapes between May 2011 and July 2013 using motion-triggered video cameras with infrared flash (Bushnell TrophyCam, Overland Park, KS). We concurrently captured and placed GPS-enabled collars (Model GPS Plus 1D, Vectronics Aerospace, Berlin, Germany) with unique identifiers on 39 pumas (females = 20, males = 19) residing in our study area. The collars were generally set to collect waypoints every 4 h, and had a mean fix rate of 76.1 (± 4.1 SE) %. The pumas with collars were monitored for a mean of 1.71 (± 0.26 SE) years. We defined community scrapes as scrape areas used for communication by >1 puma (confirmed through camera data), that were not associated with either kill or bedding sites [20]. The community scrapes we found were typically characterized by an abundance of scrapes in a small area (≥ 3 in 9m^2^) [20]. The community scrapes were also used by other carnivores for scent marking, including coyotes (*Canis latrans*), bobcat (*Lynx rufus*), gray foxes (*Urocyon cinereoargenteus*), and striped skunks (*Mephitis mephitis*). We identified areas that potentially contained community scrapes using an algorithm that identified areas visited by collared pumas multiple times but with at least 7 days in between consecutive visits (to eliminate kill sites) [21]. We also identified additional scrapes in areas not occupied by collared pumas during opportunistic site investigations. To date we have documented 299 community scrapes through field visits, and placed motion-triggered cameras at community scrapes at dispersed sites that appeared to have regular activity. We placed 1 camera at each community scrape, approximately 3 m from the scrape itself. We programmed cameras to record 60 s videos at each trigger with a 1 s delay before becoming active again in order to maximize recording time. We visited the community scrapes to maintain the cameras every 1–3 months, based on battery type and memory card capacity.

When possible, we identified the individual puma recorded in videos and placed them into demographic classes based on age (mature > 3.5 years or immature < 3.5 years) and sex (male or female). Demographic information, including mass and age, was readily available for collared individuals documented on cameras (n_male_ = 8, n_female_ = 13). We used the mass acquired from the capture closest to the visit, and determined age by measuring gum line recession during capture [23] and adjusting the age in half year increments following the initial capture date. For individuals without collars (n_male_ = 2, n_female_ = 22, n_unknown_ = 4), when possible, we identified sex and age class through the type and position of genitals and external physical characteristics [24,25]. Identifying individuals in species that lack definite markings is often unreliable e.g., [26]. We thus attempted to use what appeared to be unique spotting patterns on the inside of the upper leg or other distinct features including scarring, kinks in their tail, and old injuries [27,28]. In cases where an individual was not readily identifiable we did not use their identity in the statistical analyses. Due to the difficulty in identifying individuals, and our large number of females without collars, data on female visitation rates should be interpreted with caution.

We removed immature pumas and mature females traveling with kittens from our analyses, as they were less regular visitors and tend to act as non-participants in mating behaviours [20]. To ensure spatio-temporally independent samples and minimize pseudo-replication, we pooled the data from cameras placed at community scrapes < 1 km apart. We had one exception where the scrapes were separated by a lake and cliff that, based on GPS data, substantially increased the travel time of pumas. We removed from our analyses any community scrapes we monitored for < 3 months, and excluded any periods with camera malfunctions from our visitation samples. This resulted in 30 distinct community scrape areas monitored during this study.

We monitored these 30 community scrapes for a mean of 501 days ± 38 SE (range 125–791), and watched each video that met our previously listed criteria (676 visits by mature males and 179 visits by mature females traveling without kittens). For each visit, we recorded the date and time, length of visit to the closest second (averaged for pooled samples), and number of scrapes created (averaged for pooled samples). We also recorded events of consorting with pumas of the opposite sex, and behaviours possibly associated with mate selection, including scraping, olfactory investigating, caterwauling, and flehmen response (see Table 1 for definitions).

### Statistical analyses

We used program *R* version 3.1.0 [29] for all statistical analyses. In each analysis we considered *p* < 0.05 to represent statistical significance. The data used in these analyses is available using the doi address: doi:10.5061/dryad.6d5h2.

We first tested for differences in visitation between male and female pumas using the ‘days until next visit’ as our variable, with a mixed-model analyses of variance (ANOVAs) within the *nlme* package [30]. We measured days until next visit as the number of days for the same individual to return to the same community scrape. We compared male visitation to two categories of female visitation, first comparing male visitation to all female visitation. Second, because female visitation occurred in shorter visitation bouts, we compared male visitation to females during their visitation bouts. We defined visitation bouts as repeated visits by an individual female with ≤ 60 days between visits, based on a natural break in the data [31,32]. In our analysis we used days until next visit as our dependent variable, sex of the pumas as the independent variable, and used the individual puma id as a random effect to account for variation among individuals.

Based on GPS location data, we estimated 95% local convex hull (LoCoH) home ranges [33] using the LoCoH tool in ArcMap (v. 10.1, ESRI, 2012). We separated locations into two yearly monitoring periods (from July 2011-June 2012 and July 2012-June 2013), and included any collared adult puma with a minimum of three months of monitoring during a given period, resulting in a mean of 1120 points (± 107 SE) to calculate each home range. We then documented each foray outside of the 95% home range during each monitoring period, where we considered a foray to be the presence of 2 or more continuous GPS locations > 1 km outside of the yearly 95% LoCoH home range (mean_female_ = 37.9 km^2^ ± 3.3 SE, mean_male_ = 115.7 km^2^ ± 13.2 SE). We used a two-tailed t-test assuming equal variances to test for differences between male and female pumas, after first testing the data for normality and homoscedasticity. Finally, we determined the number of male pumas each female overlapped with during each annual monitoring period using the 95% LoCoH home ranges.

We determined which male characteristics and behaviours best predicted being selected as a mate by female pumas. We compared variables of behaviours exhibited during visitation (total number of scrapes created, mean number of scrapes created per visit, number of visits, and mean duration of visits) and physical characteristics (mass during most recent capture, and age) between two different time periods: during female visitation bouts to determine what is important while a female is actively selecting a mate, and in the 90 days previous to the consorting to determine the potential role of advertisement and other aspects prior to female arrival. We choose a 90-day period prior to mating as it coincides with the known gestation duration of pumas [8]. We included all females that consorted with ≥1 male in the analysis. We defined consorting as traveling with a male (n = 16) (S3 Video) or visiting scrapes within two hours of each other (n = 7) (where we considered it likely that the individuals met despite the event not being captured by our cameras based on associated GPS data). We used whether a male was selected as our dependent variable, and used the visitation behaviour and physical characteristics as our predictor variables. We used a stepwise AIC procedure in the *MASS* package [34] to determine the best model. The combination of all possible variables was used, and in a stepwise process each variable was eliminated until the best model based on AIC scores was arrived at for both time periods.

## Results

### Patterns in visitation

Visitation by females at community scrapes (S4 Video) occurred in short visitation bouts (n = 40) or single visits (n = 28). Visitation bouts had a mean duration of 31.8 (± 5.1 SE) days, and included a mean of 3.7 (± 0.4 SE) visits per bout. Females had a mean of 11.8 (± 1.5 SE) days between visits during a given visitation bout. The visitation bouts of females occurred throughout the year, and were separated by large gaps in time (mean = 367 ± 62 SE days).

Mature male and female pumas varied in their patterns of visitation to community scrapes. In contrast to females, visitation by males at community scrapes (S5 Video) was characterized by regular visits throughout monitoring periods that averaged 17.7 (± 2.1 SE) days between visits. Visitation of females during their visitation bouts was 1.5 times more frequent than male pumas (*F*_1, 650_ = 13.4, *p* < 0.0001), while male visitation was twice as frequent as females overall (mean = 33.2 days ± 8.9 SE, *F*_1, 657_ = 2.9, *p* = 0.0959). In essence, male pumas visited regularly throughout the year, while females visited less frequently overall, but visited more frequently during short visitation bouts.

On three occasions we documented on camera an individual male at a community scrape outside of its 95% LoCoH home range for the first time during a female visitation bout. On these occasions, the males arrived during the visitation bout, and then after the female visitation bout ended the male’s visitation to the community scrape decreased in frequency and eventually ended. This suggests that these male pumas moved out of their normal territory to take advantage of the temporal presence of a female pumas in oestrus, who possibly advertised their presence across long distances by caterwauling. We documented females exhibiting caterwauling (S2 Video) during 28.2% of visitation bouts. Our spatial analysis found that males made 6.7 (± 1.3 SE, range 2–12) forays outside of their home range during yearly monitoring periods. This was significantly more than the 2.0 (± 0.4 SE, range 0–5) annual forays made by females (*t*_18_ = 4.3, *p* = 0.0004).

### Female mate selection

**Fig 2:**
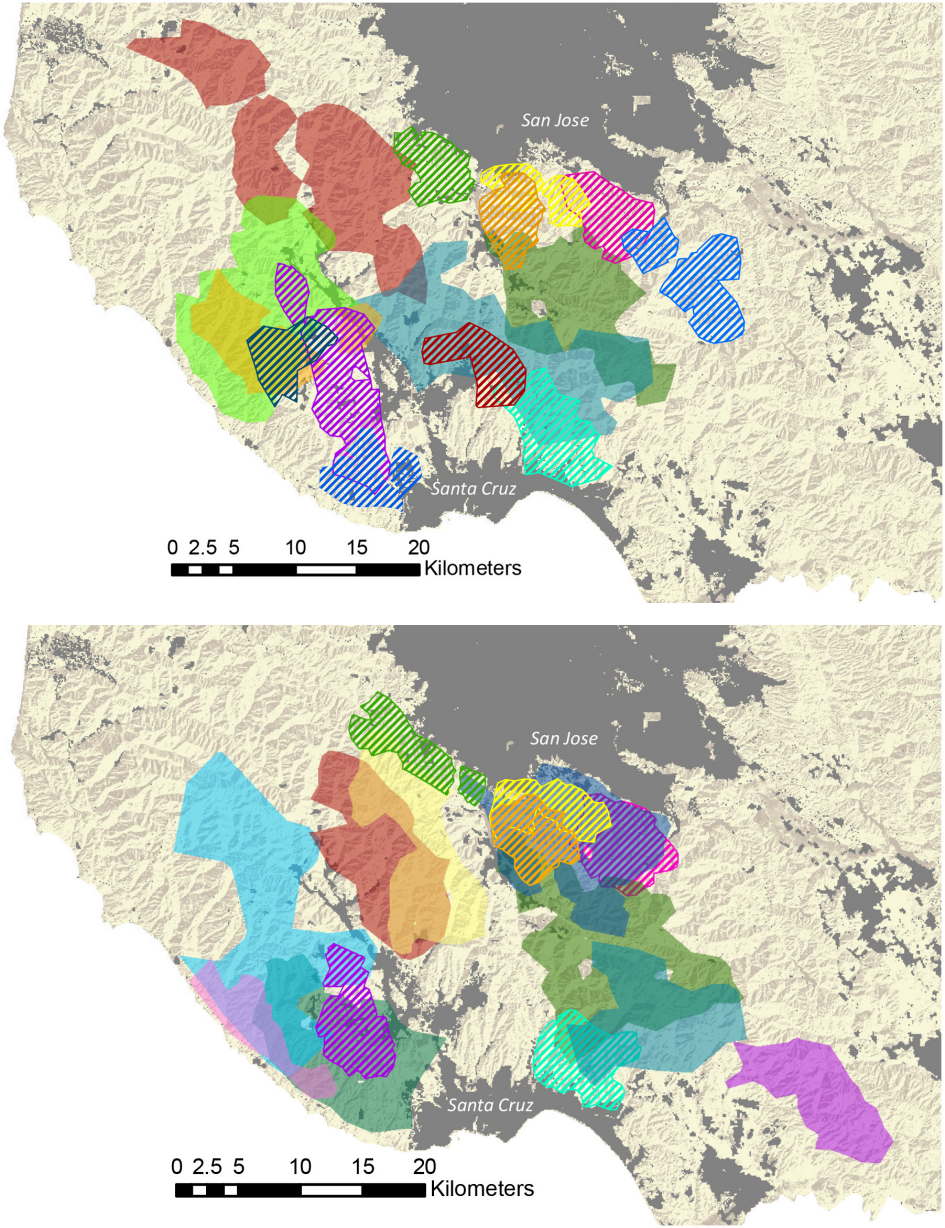
A map showing the overlap among pumas using 95% LoCoH home ranges in the study area. The upper map shows the overlap in our first time period (July 2011 to June 2012), and the lower map shows the overlap in our second time period (July 2012 to June 2013).

We documented 23 incidents of consorting with mates (Video 5) by 17 female pumas. Females who consorted with a male selected from a mean of 1.7 (range = 1–3) males based on video data. This was similar to the number of males a female’s home range overlapped with; from July 2011 to June 2012 each female monitored with GPS collars overlapped with 1.7 males with GPS collars (range 1–3), and from July 2012 to June 2013 each female monitored with GPS collars overlapped with 2.0 males with GPS collars (range = 0–3) (Fig 2). Of the 17 females we recorded consorting with a male, 2 (12%) consorted with 2 different males during a visitation bout.

All of the males selected of known age (n = 8) were between the ages of 3 to 7 years old. During a female’s visitation bout, model selection results suggested that the best model included additive effects of the number of visits, mass and age (AIC_*w*_ = 0.54), which had more than twice the support of any other models we considered (Table 2). In the time period 90 days before the consorting event, model selection results found that a single predictor variable associated with visits, the total number of scrapes created (AIC_*w*_ = 0.56), was the best model and performed twice as well as any other model we considered (Table 2).

**Table 2:** Predictors of female mate selection, represented by the best models during the female’s visitation bout, and 90 days previous to consorting with males. We determined the best models using combinations of variables^1^, and for each model we report the variables of the best model and the C-statistic score, the AIC score, and the AIC_*w*_ weight for each of the best 3 models.

Time Period	Model	C	AIC	ΔAIC	AIC_*w*_
During	VSTS + MASS + AGE	0.81	34.5	0	0.54
	VSTS + MDUR + MASS + AGE	0.8	35.8	1.3	0.28
	TSCR + VSTS + MDUR + MASS + AGE	0.8	36.7	2.2	0.18
90 days	TSCR	0.72	32	0	0.56
	TSCR + MDUR	0.81	33.5	1.5	0.27
	TSCR + MDUR + AGE	0.77	35.3	3.3	0.11

^1^ TSCR = The total number of scrapes made, MSCR = The mean number of scrapes made, VSTS = The number of visits made, MDUR = The mean duration of visits, MASS = The mass of the male, AGE = The age of the male.

## Discussion

Our objective was to increase our understanding of mate selection strategies used by female pumas based on behaviours we recorded with video cameras at community scrapes and GPS location data that allowed us to consider aspects of the spatial distribution of the population. Our results suggest sex-specific differences in mate selection strategies, with males potentially attempting to mate with every female they encounter and females actively selecting their mates. We found that females varied from males in visitation rates of and behaviours displayed at community scrapes, and appeared to use multiple cues to select mates. These results support previous suggestions that community scrapes effectively function as “billboards” e.g., [8] where individual males advertise their reproductive status and females select from potential mates. Community scrapes thus appear to play an integral role in communication and mate selection in pumas.

Female pumas apparently used multiple cues in their selection of mates, as is common in other mammals [3], and supported our hypothesis that females would select for the dominant resident males. During female visitation bouts, females selected males based on their number of visits, mass, and age. In the period 90 days previous to consorting, the total scrapes made by a male were the most important aspect of that male being selected. These patterns imply that while females prefer larger and older (>3 years) males (which are likely correlated, and an indication of dominance [8,9]), frequent community scrape visitation and scent marking can greatly improve mating opportunities for males. In addition to increasing the probability of an encounter with a female in oestrus, frequent visitation to a community scrape also serves to advertise their residency. The apparent use of these physical and temporal cues during a female’s visitation bout suggest that females may evaluate physical characteristics and dominance when selecting mates. The importance of scraping activity prior to consorting events also suggests that females select the resident, and hence dominant, male. The male puma that scrapes the most is likely to be the dominant male in the area, as juvenile pumas rarely scrape [8,20], and it is resident males who most frequently visit and scent mark in a given area. Mating with the dominant male likely reduces the risk of infanticide e.g., [8]. The use of the these multiple cues likely allows females to evaluate mate quality, reduce errors in selecting suboptimal mates, and may also allow for individuals to select traits they deem most important to the survival of their offspring e.g., [3, 35].

Female pumas differed from males in their visitation and mate selection strategies, and appeared to be influenced by a complex set of cues and behaviours at community scrapes. Our results supported our hypothesis, by indicating that although visits to community scrapes by females occurred over the entire year, visits of individuals were likely correlated with oestrus bouts, with frequent visitation occurring over short time periods that potentially overlapped with 2 oestrus cycles (mean = 32 days). Our data for female pumas was predominantly based on individuals without collars, and is hence less exact, but the irregularity in female visitation rates was in contrast to males who regularly visited community scrapes to advertise their presence through scent marking. Scrape visitation was predominately a male activity, and male pumas exhibit vigilance through regular visiting and advertising their presence for prospective mates as well as territorial rivals [8,20]. The use of community scrapes is thus an important mechanism for communication with potential mates in spatially dispersed populations of solitary felids, and could possibly be more important for males to advertise dominance in species with spatially clumped populations.

Female pumas appeared to consort with one of the dominant resident males that overlapped their home range, which were also likely the males they were most familiar with. This highlights the importance of access to females in male territoriality and its role in intraspecific competition. Mass of males is an important adaptation for competition, and is often correlated with age, and hence residency. It is an important competitive advantage in confrontations, as larger males can prevent the access of smaller males to females either through fighting or intimidation. Aggressive disputes are relatively infrequent among resident male felids due to the potential for serious injury [8,36,37], but access to reproductive females may temporarily outweigh risks associated with interactions with other males [12,37], reinforcing the importance of large mass in male solitary felids.

Although most solitary carnivores regularly visit and scent mark areas to maintain a territory, our findings suggest that attempts to exclude other males through scent marking are apparently not completely successful in spatially dispersed populations. We recorded 3 incidents on video where non-resident males consorted with females that were visiting community scrapes that previously had been used by only one male. Our analyses of forays outside of the home range documented that forays by male pumas are more common than previously thought, and were more frequent than forays by female pumas, supporting our hypothesis. Female pumas caterwauling may be the cause of these incidents of temporary territorial transgressions, as the function of caterwauling (S2 Video) is thought to be to advertise from a distance [8], and is likely a mechanism to alert males to the presence of females to which they would otherwise be unaware. Although we documented caterwauling at 28.2% of female visitation bouts, this is likely an underestimate because our cameras monitored only a small part of the area potentially used by pumas during courtship and mating. It is unknown if female pumas use caterwauling to advertise their presence to all males or a specific individual. Because males continue to scrape while consorting with a female, we expect that females are able to discern between trespassing males and territorial males that had previously been scraping. Therefore, in the instances where females select a trespassing male, the opportunity to increase the genetic diversity of their offspring may be more important than residence or familiarity.

We documented that female pumas are sometimes polyandrous, as we recorded two female pumas consorting with more than one male during visitation bouts (14.2% of the consorting documented). A genetic analysis of kittens in the study area showed a similar pattern with two sires in one of eight litters tested (12.5% of litters) (Wilmers, unpublished data). Other mammals have shown increased reproductive success associated with polyandry [38], and the same may be true of pumas. Sperm competition plays an important role in mate selection in species with confirmed polyandry [9], but there are other adaptive benefits of confusing paternity. Because many male felids, including pumas, frequently kill unrelated kittens in order to induce ovulation in females and increase their own reproductive success, mating with multiple males may decrease the probability of infanticide [8,39,40]. Polyandry also allows for greater genetic diversity in the offspring of the female, limits risk of genetic incompatibility or defects, and increases the survival probability of at least one kitten in the case of resource uncertainty [41].

## Supporting Information

S1 Video Scraping behaviour by a mature male puma. (MP4)

S2 Video A subset of a female visit, highlighting caterwauling. (MP4)

S3 Video A mature male and mature female consorting at a community scrape. (MP4)

S4 Video A female visiting a community scrape. (MP4)

S5 Video A typical male visit to a community scrape. (MP4)

## References

[1] Darwin C (1859). On the origin of species by means of natural selection. John Murray, London.

[2] Gibson RM, Langen TA (1996) How do animals choose their mates? Trends in Ecology and Evolution 11:468–470. 21237923 10.1016/0169-5347(96)10050-1

[3] Candolin U (2003) The use of multiple cues in mate choice. Biological Reviews 78:575–595. 14700392 10.1017/s1464793103006158

[4] Bailey TN (1974) Social Organization in a Bobcat Population. Journal of Wildlife Management 38:435–446.

[5] Stenstrom D, Dahlblom S, Jones Fur C, Hoglund J (2000) Rutting pit distribution and the significance of fallow deer *Dama dama* scrapes during the rut. Wildlife Biology 6:23–29.

[6] Hurst JL, Benyon RJ (2004) Scent wars: the chemobiology of competitive signalling in mice. BioEssays 26:1288–1298. 15551272 10.1002/bies.20147

[7] Stein AB, Hayssen V **(** 2013) Panthera pardus. Mammalian Species 47:30–48.

[8] Logan K, Sweanor L (2001) Desert puma: evolutionary ecology and conservation of an enduring carnivore. Island Press: 464 pages.

[9] Andersson M, Simmons LW (2006) Sexual selection and mate choice. Trends in Ecology and Evolution 21:296–302. 16769428 10.1016/j.tree.2006.03.015

[10] Gorman ML, Trowbridge BJ (1989) The role of odor in the social lives of carnivores In: Carnivore behavior, ecology, and evolution (Gittleman JG, ed), Comstock Publishing Associates, Cornell, NY, p. 57–88.

[11] Gehrt SD, Fritzell EK (1998) Resource distribution, female home range dispersion and male spatial interactions: group structure in a solitary carnivore. Animal Behavior 55:1211–1227. 10.1006/anbe.1997.0657 9632506

[12] Smith JLD, McDougal C, Miquelle D (1989) Scent marking in free—ranging tigers, Panthera tigris. Animal Behaviour 37:1–10.

[13] Clapham M, Nevin OT, Ramsey AD, Rosell F (2012) A hypothetico-deductive approach to assessing the social function of chemical signaling in a non-territorial solitary carnivore. PLoS One 7:e35404 10.1371/journal.pone.0035404 22530018 PMC3329431

[14] Bothma JP & le Riche EAN (1993) Evidence of the use of rubbing, scent marking and scratching posts by Kalahari leopards. Journal of Arid Environments 29:511–517.

[15] Seidensticker JC IV, Hornocker MG, Wiles WV, Messick JP (1973) Mountain lion social organization in the Idaho Primitive Area. Wildlife Monographs 35:3–60.

[16] Logan K, Sweanor L (2010) Behavior and social organization of a solitary carnivore In Cougar: ecology and conservation, edited by Hornocker M, Negri S. University of Chicago Press, Chicago: 105–117.

[17] Laundre JW, Hernandez L (2007) Do female pumas (*Puma concolor*) exhibit a birth pulse? Journal of Mammalogy 88:1300–1304.

[18] Jansen BD, Jenks JA (2012) Birth timing for mountain lions (*Puma concolor*); testing the prey availability hypothesis. PLoS One 7:e44625 10.1371/journal.pone.0044625 23028569 PMC3454394

[19] Harmsen BJ, Foster RJ, Gutierrez SM, Marin SY, Doncaster CP (2010) Scrape-marking behavior of jaguars (Panthera onca) and pumas (Puma concolor). Journal of Mammalogy 91:1225–1234.

[20] Allen ML, Wittmer HU, Wilmers CC (2014) Puma communication behaviours: understanding functional use and variation among sex and age classes. Behaviour 151:819–840.

[21] Wilmers CC, Wang Y, Nickel B, Houghtaling P, Shakeri Y, Allen ML, et al (2013) Scale dependent behavioural responses to human development by a large predator, the puma. PLoS One 8:e60590 10.1371/journal.pone.0060590 23613732 PMC3629074

[22] Sikes RS, Gannon WL, and the Animal Care and Use Committee of the American Society of Mammalogists (2011) Guidelines of the American Society of Mammalogists for the use of wild mammals in research. Journal of Mammalogy 92: 235–253. 10.1093/jmammal/gyw078 PMC5909806 29692469

[23] Laundre JW, Hernandez L, Streubel D, Altendorf K, Gonzalez CL (2000) Aging mountain lions using gum-line recession. Wildlife Society Bulletin 28: 963–966.

[24] Ashman D, Christensen GC, Hess ML, Tsukamoto GK, Wickersham MS (1983) The mountain lion in Nevada. Nevada Game and Fish Department, final report for project W-48-15.

[25] Currier MJP (1983) Felis concolor. Mammalian Species 200:1–7.

[26] Guthlin D, Storch I, Kuchenhoff H (2014) Is it possible to individually identify red foxes from photographs? Wildlife Society Bulletin 38:205–210.

[27] Kelly MJ, Noss AJ, DiBitetti MS, Maffei L, Arispe RL, Paviolo A, et al (2008) Estimating puma densities from camera trapping across three study sites: Bolivia, Argentina, and Belize. Journal of Mammalogy 89:408–418.

[28] McBride R, Sensor R (2015) Efficacy of trail cameras to identify individual Florida panthers. Southeastern Naturalist 14: 351–360.

[29] R Core Team (2014) R: A language and environment for statistical computing. R foundation for statistical computing, Vienna, Austria.

[30] Pinheiro J, Bates D, DebRoy S, Sarkar D (2013) nmle: linear and nonlinear mixed effect models. R package version 3.1–108.

[31] George S, Crooks K (2006) Recreation and large mammal activity in an urban nature reserve. Biological Conservation 133:107–117.

[32] Wang Y, Allen ML, Wilmers CC (2015) Mesopredator spatial and temporal responses to large predators and human development in the Santa Cruz Mountains of California. Biological Conservation 190:23–33.

[33] Getz WM, Fortmann-Roe S, Cross PC, Lyons AJ, Ryan SJ, Wilmers CC (2007) LoCoH: nonparameteric kernel methods for constructing home ranges and utilization distributions. PLoS One 2:e207 17299587 10.1371/journal.pone.0000207 PMC1797616

[34] Venables WN, Ripley BD (2002) Modern applied statistics with S. 4th edition Springer, New York.

[35] Shuett W, Tregenza T, Dall SRX (2010) Sexual selection and animal personality. Biological Reviews 85:217–246. 10.1111/j.1469-185X.2009.00101.x 19922534

[36] Enquist M, Leimar O (1990) The evolution of fatal fighting. Animal Behavior 39:1–9.

[37] Mattisson J, Segerstrom P, Perssson J, Aronsson M, Rauset GR, Samelius G, et al (2013) Lethal male—male interactions in Eurasian Lynx. Mammalian Biology 78:304–308.

[38] Hoogland JL (2013) Why do female prairie dogs copulate with more than one male?—Insights from long-term research. Journal of Mammalogy 94:731–744.

[39] Balme GA, Batchelor A, De Woronin Britz N, Seymour G, Grover M, Hes Po L, et al (2013) Reproductive Success of female leopards *Panthera pardus*: the importance of top-down processes. Mammal Review 43:221–237.

[40] Balme GA, Hunter LTB (2013) Why do leopards commit infanticide? Animal Behaviour 86:791–799.

[41] Simmons LW (2005) The evolution of polyandry: sperm competition, sperm selection, and offspring viability. Annual Review of Ecology, Evolution, and Systematics 36:125–146.

